# Temporal reciprocal relationships among anxiety, depression, and posttraumatic stress disorder for family surrogates from intensive care units over their first two bereavement years

**DOI:** 10.1186/s12888-023-04916-4

**Published:** 2023-06-08

**Authors:** Fur-Hsing Wen, Yeong-Yuh Juang, Holly G. Prigerson, Wen-Chi Chou, Chung-Chi Huang, Tsung-Hui Hu, Ming Chu Chiang, Li-Pang Chuang, Siew Tzuh Tang

**Affiliations:** 1 Department of International Business, Soochow University, Jiangsu, Taiwan, R. O. C.; 2 Department of Psychiatry, Koo Foundation Sun Yat-Sen Cancer Center, Taipei, Taiwan, R. O. C.; 3 Department of Medicine, Weill Cornell Medicine, New York City, NY USA; 4 Division of Hematology-Oncology, Chang Gung Memorial Hospital at Linkou, Tao-Yuan, Taiwan, R. O. C.; 5 College of Medicine, Chang Gung University, Tao-Yuan, Taiwan, R. O. C.; 6 Department of Internal Medicine, Division of Pulmonary and Critical Care Medicine, Chang Gung Memorial Hospital at Linkou, Tao-Yuan, Taiwan, R. O. C.; 7 Department of Respiratory Therapy, Chang Gung University, Tao-Yuan, Taiwan, R. O. C.; 8 Department of Internal Medicine, Division of Hepato-Gastroenterology, Chang Gung Memorial Hospital at Kaohsiung, Kaohsiung, Taiwan, R. O. C.; 9 Department of Nursing, Chang Gung Memorial Hospital at Kaohsiung, Kaohsiung, Taiwan, R. O. C.; 10 School of Nursing, Medical College, Chang Gung University, 259 Wen-Hwa 1st Road, Kwei-Shan, 333 Tao-Yuan, Taiwan, R. O. C.; 11 Department of Nursing, Chang Gung University of Science and Technology, Tao-Yuan, Taiwan, R. O. C.

**Keywords:** Anxiety, Depression, PTSD, Comorbidity, Temporal relationships, ICU care, End-of-life care, Family members

## Abstract

**Background/Objective:**

Bereaved family surrogates from intensive care units (ICU) are at risk of comorbid anxiety, depression, and post-traumatic stress disorder (PTSD), but the temporal reciprocal relationships among them have only been examined once among veterans. This study aimed to longitudinally investigate these never-before-examined temporal reciprocal relationships for ICU family members over their first two bereavement years.

**Methods:**

In this prospective, longitudinal, observational study, symptoms of anxiety, depression, and PTSD were assessed among 321 family surrogates of ICU decedents from 2 academically affiliated hospitals in Taiwan by the anxiety and depression subscales of the Hospital Anxiety and Depression Scale, and the Impact of Event Scale-Revised, respectively at 1, 3, 6, 13, 18, and 24 months postloss. Cross-lagged panel modeling was conducted to longitudinally examine the temporal reciprocal relationships among anxiety, depression, and PTSD.

**Results:**

Examined psychological-distress levels were markedly stable over the first 2 bereavement years: autoregressive coefficients for symptoms of anxiety, depression, and PTSD were 0.585–0.770, 0.546–0.780, and 0.440–0.780, respectively. Cross-lag coefficients showed depressive symptoms predicted PTSD symptoms in the first bereavement year, whereas PTSD symptoms predicted depressive symptoms in the second bereavement year. Anxiety symptoms predicted symptoms of depression and PTSD at 13 and 24 months postloss, whereas depressive symptoms predicted anxiety symptoms at 3 and 6 months postloss while PTSD symptoms predicted anxiety symptoms during the second bereavement year.

**Conclusions:**

Different patterns of temporal relationships among symptoms of anxiety, depression, and PTSD over the first 2 bereavement years present important opportunities to target symptoms of specific psychological distress at different points during bereavement to prevent the onset, exacerbation, or maintenance of subsequent psychological distress.

**Supplementary Information:**

The online version contains supplementary material available at 10.1186/s12888-023-04916-4.

## Introduction

Death and dying in intensive care units (ICUs) is common [1, 2] and increasing [3] worldwide and even more so given COVID-19 deaths often occur in ICUs [4]. The uncertain terminal trajectory of critical illness, the frightening nature of aggressive, life-prolonging care that threatens the patient’s bodily integrity [5], and ultimately the loved one’s frequently unexpected death predispose family members to increased risks for post-intensive care syndrome (PICS-F) [6] during bereavement, including symptoms of anxiety [7–16], depression [7–20], and post-traumatic stress disorder (PTSD) [7, 9–21]. Grief reactions may reciprocally interfere with one another [22]. Psychological distress commonly co-occurs as depression and anxiety [23]; depression and PTSD [24]; or anxiety, depression, and PTSD [25] to synergistically impair physical and mental-health functioning [23, 24, 26, 27], survival [28], and treatment outcomes [29]. Therefore, recognizing the potential of comorbid psychological distress and identifying modifiable risks precipitating their co-occurrence, trajectories, and future onset is paramount for improving psychological well-being of ICU decedents’ family members.

One potential mechanism for comorbid psychological distress is that preexisting psychological distress increases the risk of subsequent other psychological distress [30]. Knowledge about the temporal relationships among anxiety, depression, and PTSD elucidates how comorbidity evolves and has important clinical implications for prevention or treatments/management of those types of psychological distress [30, 31]. However, only one study was found to investigate the temporal reciprocal relationships on how symptoms of anxiety, depression, and PTSD influence each other over time among war veterans [25]. Therefore, the purpose of this study was to longitudinally determine the temporal reciprocal relationships among symptoms of anxiety, depression, and PTSD of ICU decedents’ family surrogates over their first two bereavement years to examine the potential mechanism of comorbidity of these three types of psychological distress.

## Materials and methods

### Study design/setting/study participants

This study is part of a longitudinal, observational study on associations between quality of end-of-life care in ICUs and family surrogates’ bereavement outcomes, including symptoms of anxiety [32], depression [32], and PTSD [33]. Sampling strategy and characteristics of the study settings were reported [32, 33]. Family surrogates of critically ill patients at high risk of death (APACHE II scores > 20) [34] were recruited consecutively from level III medical ICUs staffed by intensivists in two academically affiliated hospitals in Taiwan from January 2018 to March 2020 and followed through December 2021. Family surrogates were defined as those who self-identified as legally authorized to be the patient’s surrogate for his/her medical decisions. Each surrogate signed informed consent for participation and for allowing review of the patient’s medical record. This study was approved by the research ethics committee of the study site (Chang Gung Memorial Foundation, Institute Review Board, 201700343B0).

### Data collection

Participant demographics and preexisting comorbidities were recorded at enrollment. Surrogates were phone interviewed to assess their psychological distress at 1, 3, 6, 13, 18, and 24 months postloss to comply with the duration criterion for PTSD ≥ 1 month [35] and to avoid measuring the anniversary effect at 12 months postloss.

### Measures

*Anxiety and depressive symptoms* were measured by the Hospital Anxiety and Depression Scale (HADS) [36]. Seven HADS items measure anxiety (HADS-A subscale) and depression (HADS-D subscale), respectively, and each has a total score ranging from 0 to 21.

*PTSD symptoms* were measured by the 22-item Impact of Event Scale-Revised (IES-R) [35]. The IES-R cannot diagnose PTSD but acts as a screening instrument for PTSD symptoms and is widely used with ICU bereaved family members [7, 9, 11–13, 19–21]. The IES-R has three subscales: intrusion, avoidance, and hyperarousal. Each item is rated for its PTSD-related symptom-distress level during the preceding week on a 0 (not at all)-4 (extremely) Likert scale.

### Data analysis

To examine the temporal reciprocal relationship between anxiety, depression, and PTSD longitudinally, we conducted a cross-lagged panel modeling (CLPM; also known as autoregressive cross-lagged modeling) [37] to simultaneously assess whether the prior measure of each psychological-distress type predicts the subsequent measure of the same construct, and whether the prior measure of specific psychological distress (e.g., symptoms of PTSD) predicts the subsequent measurement of the other two psychological-distress types (e.g., symptoms of anxiety and depression) in the first two bereavement years by structural equation modeling in Mplus 8.6. CLPM is one of the most popular approaches for addressing temporal reciprocal relationships in medical research [38]. In CLPM, two relations among constructs across time are of interest [37, 39]: the autoregressive and cross-lagged relations. The autoregression component of the CLPM indicates the extent to which the later measure of a construct is predicted by an immediate earlier measure of the same construct to provide information on the stability of that specific construct; whereas the cross-lagged component denotes to what extent the prior scores of one variable relate to subsequent scores of the other variables. The advantage of analyzing cross-lagged effects is to assess the strength of prediction among variables (cross-lag coefficients) while controlling for each variable’s immediate prior time score (autoregressive coefficients) to inform temporal precedence (or causal relationship) among constructs across time [39]. Of note, despite the advantages of using CLPM to illustrate temporal reciprocal relationships among symptoms of anxiety, depression, and PTSD, the CLPM is criticized as unable to conclusively address the reciprocal relationships because it cannot distinguish within- and between-person sources of variance [37]. However, the goal of modeling development processes (as within a personal growth pattern) should be kept separate from the goal of causal inference (as temporal relationships between variables). If the goal is to examine the causal inference of temporal reciprocal relationships between variables as in this study, use of traditional CLPM by estimating cross-lagged effects is still recommended [40]. Model fit was assessed by the following fit indices: [1] comparative fit index (CFI) > 0.90 [41], [2] Tucker-Lewis index (TLI) > 0.90 [41], [3] root mean square error of approximation (RMSEA) < 0.10 [42], and [4] standardized root mean square residual (SRMR) < 0.10 [43].

## Results

### Participant characteristics

**Fig. 1:**
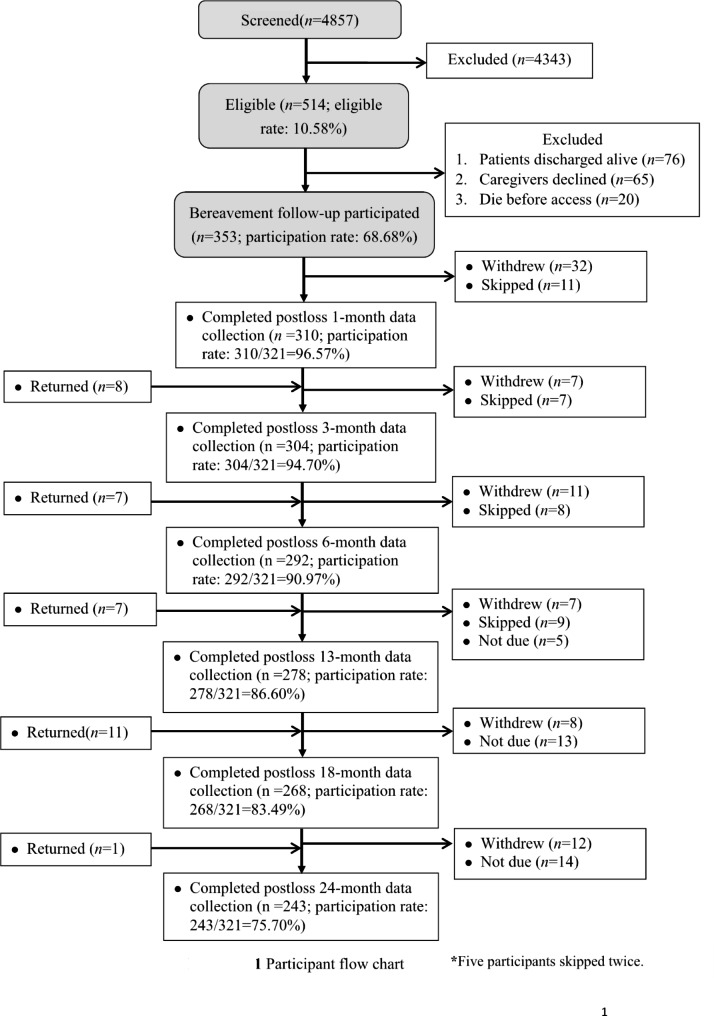
Case flow chart

Among the 353 patients who died in the ICUs, 321 family surrogates (90.9%) participated in bereavement surveys and constituted the study sample; 310, 304, 292, 278, 268, and 243 surrogates completed surveys at 1, 3, 6, 13, 18, and 24 months post-loss, respectively (Fig. 1). Detailed reasons for attrition from bereavement surveys are in Fig. 1. Patients of family surrogates who declined participation in (Table 1), skipped, or withdrew from bereavement surveys (Supplemental Table 1) did not differ by sociodemographics or clinical characteristics from patients of family surrogates who completed all assessments. Enrolled family surrogates who declined participation in (Table 2), skipped, or withdrew from bereavement surveys did not differ by sociodemographics or clinical characteristics (Supplemental Table 2) nor by prior wave of psychological distress (Supplemental Table 3) from those who completed all assessments. Characteristics of patients, the 321 study participants, and participant assessments of their psychological distress during bereavement are in Tables 1 and 2 and Supplemental Tables 3, respectively.

**Table 1:** Patient characteristics (*N* = 353)

Variable, *n* (%)	Participants (*n* = 321)	Declined bereavement surveys (*n* = 32)	*P*
Gender			0.721
Male	205 (63.9%)	22 (68.8%)	
Female	116 (36.1%)	10 (31.3%)	
Diagnosis			0.650
Cancer	160 (49.8%)	20 (62.5%)	
Chest	22 (6.9%)	2 (6.3%)	
Cardiovascular Digestive Kidney Other	15 (4.7%) 11 (3.4%) 16 (5.0%) 97 (30.2%)	2 (6.3%) 1 (3.1%) 2 (6.3%) 5 (15.6%)	
Acute symptoms/problems at admission			0.160
Respiratory failure/distress Infection Shock Bleeding Cardiac arrest Others	167 (52.0%) 91 (28.3%) 24 (7.5%) 10 (3.1%) 12 (3.7%) 17 (5.3%)	16 (50.0%) 8 (25.0%) 1 (3.1%) 2 (6.3%) 0 (0.0%) 5 (15.6%)	
Comorbidity			0.911
Yes	273 (85.0%)	28 (87.5%)	
No	48 (15.0%)	4 (12.5%)	
Variable, Mean (SD)			
Age (years)	66.78(14.26)	64.94 (9.68)	0.336
APACHE^a^	28.36(5.37)	28.28 (5.93)	0.942
SOFA^a^	12.33(4.02)	12.66 (4.23)	0.679
Length of ICU stay (days)	21.13(15.06)	21.10 (15.27)	0.989
Time from ICU admission to enrollment (days)	14.90 (12.52)	13.13 (8.49)	0.289
Time from enrollment to death (days)	7.23 (8.34)	8.97 (11.33)	0.405

a: measured at the time of enrollment

**Table 2:** Family caregiver characteristics at enrollment (*N* = 353)

Variable	Participants (*n* = 321)	Declined bereavement surveys (*n* = 32)	*P*
Age, *n* (%)			0.158
21–45	126 (39.3%)	18 (56.3%)	
46–55	89 (27.7%)	9 (28.1%)	
56–65	66 (20.6%)	4 (12.5%)	
> 65	40 (12.4%)	1 (3.1%)	
Gender, *n* (%)			0.835
Male	189 (58.9%)	20 (62.5%)	
Female	132 (41.1%)	12 (37.5%)	
Marital status, *n* (%)			0.255
Single	69 (21.5%)	10 (31.3%)	
Married/Cohabiting	242 (75.4%)	20 (62.5%)	
Separated/Widowed	10 (3.1%)	2 (6.2%)	
Educational level, *n* (%)			1.000
>High school	262 (81.6%)	26 (81.2%)	
≦High school	59 (18.4%)	6 (18.8%)	
Financial status, *n* (%)			0.153
Making ends meet	270 (84.1%)	23 (71.9%)	
Financial strain	44 (13.7%)	7 (21.9%)	
Other	7 (2.2%)	2 (6.2%)	
Relationship, *n* (%)			0.135
Spouse	94 (29.3%)	7 (21.9%)	
Child	175 (54.5%)	23 (71.9%)	
Other	52 (16.2%)	2 (6.2%)	
Chronic disease, *n* (%)			0.197
Yes	112 (34.9%)	7 (21.9%)	
No	209 (65.1%)	25 (78.1%)	
Living with the patient, *n* (%)			0.994
Yes	213 (66.4%)	20 (64.5%)	
No	108 (33.6%)	11 (35.5%)	

### Longitudinal relationships between symptoms of anxiety, depression, and PTSD

**Fig. 2:**
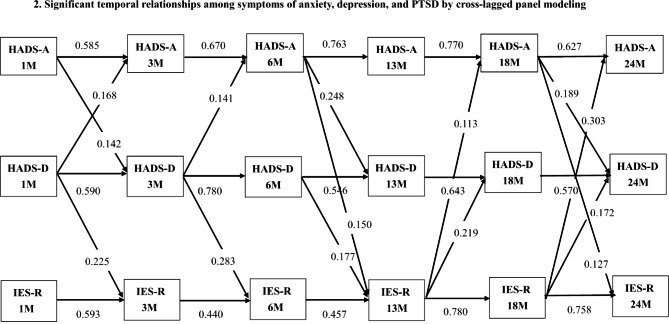
Significant temporal relationships among symptoms of anxiety, depression, and PTSD by cross-lagged panel modeling. Anxiety and depressive symptoms were measured with the anxiety (HADS-A) and depression (HADS-D) subscale of the Hospital Anxiety and Depression scale. PTSD symptoms were measured by the Impact of Event Scale-Revised (IES-R). Autoregressive coefficients along horizontal arrows indicate stability of each variable, and cross-lagged coefficients along diagonal arrows indicate strength of temporal relationships between symptoms with autoregressive coefficient controlled

The model fit the data fairly well: CFI = 0.955, TLI = 0.924, RMSEA = 0.097, and SRMR = 0.067. Analyses of the autoregressive coefficients showed that examined psychological-distress levels were markedly stable over the first 2 bereavement years (Fig. 2): autoregressive coefficients for symptoms of anxiety, depression, and PTSD were 0.585–0.770, 0.546–0.780, and 0.440–0.780, respectively.

Analyses of the cross-lagged coefficients showed symptoms of depression consistently predicted symptoms of PTSD above and beyond the autoregressive effects over the first bereavement year, but not vice versa (Fig. 2). However, an opposite relationship was shown for the second bereavement year: symptoms of PTSD consistently predicted symptoms of depression, but not vice versa. Further, symptoms of anxiety predicted symptoms of PTSD at 13 and 24 months postloss, whereas symptoms of PTSD consistently predicted symptoms of anxiety during the second bereavement year. Symptoms of PTSD and anxiety are bidirectionally related between 18 and 24 months postloss.

For the reciprocal relationships between symptoms of anxiety and depression, analyses of the cross-lagged coefficients showed that symptoms of depression only predicted symptoms of anxiety at 3 and 6 months postloss (Fig. 2), whereas symptoms of anxiety predicted symptoms of depression at 3, 13, and 24 months postloss. Symptoms of anxiety and depression are bidirectionally related between 1 and 3 months postloss.

## Discussion

We observed that examined psychological-distress levels were markedly stable over the first 2 bereavement years in line with a report for war veterans over their first two years post war [25]. Further, we found different patterns of temporal relationships between symptoms of depression and PTSD over the first 2 bereavement years. Depressive symptoms shortly postloss predicted PTSD symptoms over the first bereavement year, whereas long-lasting PTSD symptoms predicted prolonged depressive symptoms beyond the first bereavement year. The latter result was supported by a report [25] that PTSD at 1 year post war predicted depression at 2 years post war among veterans, but not vice versa. Furthermore, Glad and colleagues [44] found that PTSD symptoms within the first bereavement year do not predict later grief, whereas beyond this first year, more pervasive PTSD symptoms predict grief at 30–32 months postloss. Both studies [25, 44] confirmed that long-lasting PTSD symptoms disrupt the normal grief process and precipitate prolonged depressive (grief) symptoms beyond the first bereavement year.

The temporal relationships among psychological-distress symptoms observed in this study can be understood through the theoretical frameworks of multidimensional grief theory, stress sensitivity, and the internalizing dimension of PTSD. Multidimensional grief theory characterizes grief reactions as adaptive or maladaptive responses across three content domains: separation distress, existential/identity distress, and circumstance-related distress [22, 45]. Separation distress is characterized by missing the deceased, sadness from persistent separation, and sorrow over the deceased’s failure to physically reunite with oneself. Losing a longstanding relationship during ICU care and starting a future without the patient trigger separation distress and predispose family surrogates to suffering depressive symptoms and separation anxiety.

Because these grief reactions typically recede over time [46], we draw from the theory of stress sensitivity [47] to explain why subsequent PTSD symptoms emerge among individuals with symptoms of depression. Stress sensitivity proposes that individuals vulnerable to psychopathology may be more psychologically sensitive to stress and more susceptible to perceive stressful events as highly traumatic. Therefore, the psychological struggle among depressed family surrogates shortly after the death of their loved one may sensitize them to heightened negative responses towards proximal stressors during bereavement like loss reminders (i.e., cues that evoke memories of the deceased, their eternal absence) and traumatic memories of the ICU-care experience [48]. Sensitization to bereavement stressors may explain PTSD symptoms ensuing from depressive symptoms over the first bereavement year.

These PTSD symptoms may remain stable during bereavement due to maladaptive manifestations of separation distress [22, 45]. Separation distress has been theorized to manifest from loss reminders and to be exacerbated by maladaptive coping with loss reminders [45]. As an example of maladaptive coping, excessive behavioral or cognitive avoidance of loss reminders can interfere with remembering, reminiscing, and accepting the reality of the patient’s death [48], leading to long-lasting PTSD symptoms beyond the first bereavement year.

Co-occurrence of PTSD with major depressive and anxiety disorders has been explained by the internalizing dimension of PTSD [49]. Internalized trauma may also explain why PTSD symptoms precede subsequent anxiety and depressive symptoms in the second bereavement year. When bereaved family surrogates suffer from long-lasting PTSD symptoms, they may internalize their traumatic bereavement to manifest the existential/identity distress proposed by multidimensional grief theory [22, 45]. In other words, they may feel excessive guilt about the death, blame themselves for the occurrence of the death (self-denigration), and may not find meaning in the patient’s death, leading to negative beliefs about themselves, hopelessness, despair, a sense of a future blighted by the death, or a disparaged worldview [22, 45]. Therefore, when bereaved family surrogates internalize their long-lasting PTSD symptoms, they may suffer prolonged depressive symptoms subsequently in the second bereavement year. The same speculation can be applied to the associations between the prior wave of PTSD symptoms with subsequent heighted anxiety symptoms (e.g., like distress, disgust, and anger) in the second bereavement year.

We found that symptoms of anxiety in the prior wave of assessment consistently predicted symptoms of depression and PTSD at the first and second anniversary of bereavement (Fig. 2). For bereaved with anxiety symptoms [50], loss reminders as the anniversary of the patient’s death approached may evoke circumstance-related distress, or intrusive distressing memories of the traumatic circumstances under which their loved one died [22, 45]. Marked avoidance of such distressing intrusive memories of the loss may be elicited. Circumstance-related distress also involves feelings of helplessness, guilt, regret, or anger over not being able to save their loved one at the time of his/her death or the horrific circumstances under which the patient died. Exposure to loss reminders near the anniversary of the patient’s death also evokes bereaved caregivers’ separation distress [22, 45] to trigger their depressive symptoms as previously discussed. Therefore, bereaved family surrogates who had higher symptoms of anxiety in the prior wave of assessment [49] suffered higher depressive and PTSD symptoms in the subsequent anniversary of bereavement.

Symptoms of depression in the prior wave of assessment predicted symptoms of anxiety at both 3 and 6 months postloss during the first bereavement year, whereas symptoms of anxiety predicted symptoms of depression at 3 months postloss only. Therefore, a bidirectional relationship between symptoms of anxiety and depression was observed between 1 and 3 months postloss. Our results confirmed the conclusion from a meta-analysis that anxiety and depression are bidirectional risk factors for one another [51] at early bereavement. Furthermore, symptoms of depression at 3 months postloss continuously predicted symptoms of anxiety at 6 months postloss. As previously highlighted, the psychological struggle among depressed family surrogates shortly after the death of their loved one may sensitize them [46] to a wide range of negative emotional responses like fear, distress, disgust, and anger [52] toward the patient’s dying process and the permanent separation from their beloved, thereby suffering heighted symptoms of anxiety.

Several limitations of our study are acknowledged. Whether our findings can be generalized to (inter)national populations beyond the sampled hospitals, family surrogates of ICU patients who died within 3 days of admission, or family surrogates who did not participate in or withdrew from bereavement surveys warrants further validation. Measuring symptoms of anxiety, depression, and PTSD by screening rather than diagnostic tools likely overestimates bereaved family surrogates’ psychological distress but avoids overlooking their need for emotional support. Factors that are associated with, mediate, or moderate the interrelationships between symptoms of anxiety, depression, and PTSD have not yet been explored. Further investigation is warranted.

## Conclusion

Symptoms of anxiety, depression, and PTSD are highly stable over the first 2 bereavement year. Symptoms of depression shortly after the death of a loved one predict symptoms of PTSD in the first bereavement year, whereas long-lasting symptoms of PTSD interfere with the grief process leading to prolonged symptoms of anxiety and depression at the second bereavement year. Heightened anxiety symptoms in the previous wave of assessment predict symptoms of depression and PTSD at the subsequent first and second anniversaries of bereavement. With the remarkably high stability of anxiety, depression, and PTSD symptoms over the first 2 bereavement years, appearance of psychological distress shortly after the patient’s death should alert healthcare professionals to prevent persistent psychological suffering over time. Knowledge about the temporal relationships among symptoms of anxiety, depression, and PTSD has important implications for healthcare professionals working with bereaved family members of ICU decedents: targeting symptoms of specific psychological distress at different points during bereavement may prevent the development, exacerbation, or maintenance of subsequent psychological distress. This knowledge supplements our previous conclusions that improving quality of end-of-life care in ICUs might mitigate bereaved family surrogates’ symptoms of anxiety [32], depression [32], and PTSD [33].

## Electronic supplementary material

Below is the link to the electronic supplementary material.

**Supplementary Material 1: Supplemental Table 1**. Comparisons of patient characteristics across participation status during bereavement follow-ups (*N* = 289)^a^. **Supplemental Table 2**. Comparisons of family characteristics across participation status during bereavement follow-ups (*N* = 289)^a^. **Supplemental Table 3**. Comparisons of psychological distress of family characteristics across participation status during bereavement follow-ups (*N*=321)^a^

## Data Availability

The sharing of anonymized data from this study is restricted due to ethical and legal constrictions. Data contains sensitive personal health information, which is protected under The Personal Data Protection Act, thus making all data requests subject to Institutional Review Board (IRB) approval. Per Chang Gung Memorial Hospital (CGMH) IRB, the data that support the findings of this study are restricted for transmission to those outside the primary investigative team. Data sharing with investigators outside the team requires IRB approval. All requests for anonymized data will be reviewed by the research team and then submitted to the CGMH IRB for approval. Any requests for data should be directed to Dr Siew Tzuh Tang at sttang@mail.cgu.edu.tw.

## Code availability

Specifications for MPlus codes for statistical analyses are available from the first and corresponding authors upon reasonable request.

## List of abbreviations

APACHE: Acute Physiology and Chronic Health Evaluation
CFI: Comparative fit index
CI: Confidence interval
CLPM: Cross-lagged panel modeling
HADS: Hospital Anxiety and Depression Scale
HADS-A: HADS anxiety subscale
HADS-D: HADS depression subscale
ICU: Intensive care unit
IES-R: Impact of Event Scale-Revised
PTSD: Post-traumatic stress disorder
RMSEA: Root mean square error of approximation
SRMR: Standardized root mean square residual
TLI: Tucker-Lewis index
