# Supplementary material for: Temporal reciprocal relationships among anxiety, depression, and posttraumatic stress disorder for family surrogates from intensive care units over their first two bereavement years
Source: BMC Psychiatry. 2023 Jun 8;23:412. doi: 10.1186/s12888-023-04916-4 (PMC10248341; doi:10.1186/s12888-023-04916-4)
Supplement: Supplementary file 1 — Supplementary Material 1: Supplemental Table 1. Comparisons of patient characteristics across participation status during bereavement follow-ups (N = 289)a. Supplemental Table 2. Comparisons of family characteristics across participation status during bereavement follow-ups (N = 289)a. Supplemental Table 3. Comparisons of psychological distress of family characteristics across participation status during bereavement follow-ups (N=321)a [file 12888_2023_4916_MOESM1_ESM.docx]

**Supplemental Table 1. Comparisons of patient characteristics across participation status during bereavement follow-ups (*N* = 289)^a^**

| Variable, *n* (%) | Participants  (*n* =225) | Skipped follow-ups  (*n* =19) | | Withdrew from follow-ups (*n*=45) | | *P* | | |
| --- | --- | --- | --- | --- | --- | --- | --- | --- |
| Gender |  |  | |  | | .581 | | |
| Male | 141 (62.7%) | 14 (73.7%) | | 30 (66.7%) | |  | | |
| Female | 84 (37.3%) | 5 (26.3%) | | 15 (33.3%) | |  | | |
| Diagnosis |  |  | |  | | .509 | | |
| Cancer | 114 (50.7%) | 7 (36.8%) | | 21 (46.7%) | |  | | |
| Chest | 14 (6.2%) | 3 (15.8%) | | 1 (2.2%) | |  | | |
| Cardiovascular  Digestive  Kidney  Other | 9 (4.0%)  10 (4.4%)  13 (5.8%)  65 (28.9%) | 2 (10.5%)  - (0.0%)  1 (5.3%)  6 (31.6%) | | 2 (4.4%)  1 (2.2%)  2 (4.4%)  18 (40.0%) | |  | | |
| Acute symptoms/problems at admission | | |  | |  | | .433 |  |
| Respiratory failure/distress  Infection  Shock  Bleeding  Cardiac arrest  Others | 112 (49.8%)  62 (27.6%)  23 (10.2%)  9 (4.0%)  7 (3.1%)  12 (5.3%) | 10 (52.6%)  8 (42.1%)  - (0.0%)  - (0.0%)  - (0.0%)  1 (5.3%) | | 27 (60.0%)  11 (24.4%)  1 (2.2%)  1 (2.2%)  3 (6.7%)  2 (4.4%) | |  | | |
| Comorbidity |  |  | |  | | .556 | | |
| Yes | 192 (85.3%) | 17 (89.5%) | | 36 (80.0%) | |  | | |
| No | 33 (14.7%) | 2 (10.5%) | | 9 (20.0%) | |  | | |
| Variable, Mean (SD) |  |  | |  | |  | | |
| Age (years) | 66.05 (14.24) | 71.42 (15.24) | | 66.47 (13.99) | | .291 | | |
| APACHE^b^ | 28.37 (5.21) | 25.42 (6.31) | | 27.98 (5.57) | | .070 | | |
| SOFA^b^ | 12.38 (3.84) | 11.11 (5.16) | | 12.07 (3.83) | | .337 | | |
| Length of ICU stay, days | 20.70 (15.06) | 26.31 (14.87) | | 19.80 (10.64) | | .228 | | |
| Time from ICU admission to enrollment, days | 14.69 (13.18) | 17.05 (12.41) | | 13.67 (8.19) | | .613 | | |
| Time from enrollment to death, days | 7.01 (7.76) | 10.26 (11.07) | | 7.13 (7.57) | | .234 | | |

^a^Among the 321 family surrogates who participated in bereavement surveys, follow-up assessments were not due for 32 participants. ^b^measured at enrollment.

**Supplemental Table 2.** **Comparisons of family characteristics across participation status during bereavement follow-ups (*N* = 289)^a^**

| Variable | Participants  (*n*=225) | Skipped follow-ups  (*n*=19) | Withdrew from follow-ups (*n*=45) | *P* |
| --- | --- | --- | --- | --- |
| Age, *n* (%) |  |  |  | .419 |
| 21-45 | 93 (41.3%) | 6 (31.6%) | 16 (35.6%) |  |
| 46-55 | 57 (25.3%) | 8 (42.1%) | 13 (28.9%) |  |
| 56-65 | 44 (19.6%) | 5 (26.3%) | 11 (24.4%) |  |
| >65 | 31 (13.8%) | 0 (0.0%) | 5 (11.1%) |  |
| Gender, *n* (%) |  |  |  | .009 |
| Male | 98 (43.6%) | 11 (57.9%) | 10 (22.2%) |  |
| Female | 127 (56.4%) | 8 (42.1%) | 35 (77.8%) |  |
| Marital status, *n* (%) |  |  |  | .591 |
| Single | 46 (20.4%) | 4 (21.1%) | 10 (22.2%) |  |
| Married/Cohabiting | 173 (76.9%) | 15 (78.9%) | 32 (71.1%) |  |
| Separated/Widowed | 6 (2.7%) | - (0.0%) | 3 (6.7%) |  |
| Educational level, *n* (%) |  |  |  | .857 |
| >High school | 186 (82.7%) | 15 (78.9%) | 36 (80.0%) |  |
| ≦High school | 39 (17.3%) | 4 (21.1%) | 9 (20.0%) |  |
| Financial status, *n* (%) |  |  |  | .129 |
| Making ends meet | 191 (84.9%) | 17 (89.4%) | 32 (71.1%) |  |
| Financial strain | 30 (13.3%) | 1 (5.3%) | 11 (24.4%) |  |
| Other | 4 (1.8%) | 1 (5.3%) | 2 (4.4%) |  |
| Relationship, *n* (%) |  |  |  | .314 |
| Spouse | 68 (30.2%) | 3 (15.8%) | 13 (28.9%) |  |
| Child | 116 (51.6%) | 14 (73.7%) | 27 (60.0%) |  |
| Other | 41 (18.2%) | 2 (10.5%) | 5 (11.1%) |  |
| Chronic disease, *n* (%) |  |  |  | .627 |
| Yes | 84 (37.3%) | 5 (26.3%) | 16 (35.6%) |  |
| No | 141 (62.7%) | 14 (73.7%) | 29 (64.4%) |  |
| Living with the patient, *n* (%) | |  |  | .784 |
| Yes | 148 (65.8%) | 11 (57.9%) | 29 (64.4%) |  |
| No | 77 (34.2%) | 8 (42.1%) | 16 (35.6%) |  |

| Variable | Participants  (*n*=225) | Skipped follow-ups  (*n*=19) | Withdrew from follow-ups (*n*=45) | *P* |
| --- | --- | --- | --- | --- |
| Hospitalization for mental health problems, *n* (%) | | | | .^b^ |
| Yes | 0 (0.0%) | 0 (0.0%) | 0 (0.0%) |  |
| No | 224 (100.0%) | 19 (100.0%) | 45 (100.0%) |  |
| Hospitalization for medical problems, *n* (%) | | | | .723 |
| Yes | 11 (4.9%) | 1 (5.3%) | 1 (2.2%) |  |
| No | 214 (95.1%) | 18 (94.7%) | 44 (97.8%) |  |
| Emergency room visit, *n* (%) | |  |  | .631 |
| Yes | 18 (8.0%) | 2 (10.5%) | 2 (4.4%) |  |
| No | 207 (92.0%) | 17 (89.5%) | 43 (95.6%) |  |
| Medication use for pain problems, *n* (%) | | |  | .137 |
| Yes | 31 (13.8%) | 1 (5.3%) | 2 (4.4%) |  |
| No | 194 (86.2%) | 18 (94.7%) | 43 (95.6%) |  |
| Medication use for anxiety problems, *n* (%) | | |  | .780 |
| Yes | 6 (3.7%) | 1 (5.3%) | 1 (2.2%) |  |
| No | 219 (97.3%) | 18 (94.7%) | 44 (97.8%) |  |
| Medication use for depressive problems or other psychiatric disturbances, *n* (%) | | | | .751 |
| Yes | 2 (0.9%) | 0 (0.0%) | 0 (0.0%) |  |
| No | 223 (99.1%) | 19 (100.0%) | 45 (100.0%) |  |

^a^Among the 321 family surrogates who participated in bereavement surveys, follow-up assessments were not due for 32 participants.

^b^ cannot be estimated.

**Supplemental Table 3. Comparisons of psychological distress of family characteristics across participation status during bereavement follow-ups (*N* =321)^a^**

| Variable |  | Participants |  | Skipped follow-ups |  | Withdrew from follow-ups | *P* |
| --- | --- | --- | --- | --- | --- | --- | --- |
| HADSA, mean (SD) | n^b^ |  | n |  | n |  |  |
| Time 3 | 296 | 5.19(3.88) | 7 | 4.14(4.63) | 7 | 5.43(3.31) | 0.768 |
| Time 6 | 285 | 3.89(3.64) | 8 | 3.63(2.93) | 11 | 2.00(1.18) | 0.226 |
| Time 13 | 271 | 2.68(2.70) | 9 | 2.78(1.86) | 7 | 1.71(1.38) | 0.654 |
| Time 18 | 257 | 2.23(2.60) |  |  | 8 | 1.88(1.81) | 0.702 |
| Time 24 | 241 | 2.11(2.22) |  |  | 12 | 3.17(2.89) | 0.113 |
| HADSD, mean (SD) |  |  |  |  |  |  |  |
| Time 3 | 296 | 7.09(4.36) | 7 | 4.86(3.34) | 7 | 7.00(4.51) | 0.406 |
| Time 6 | 285 | 5.39(4.10) | 8 | 3.50(2.20) | 11 | 3.27(3.07) | 0.109 |
| Time 13 | 271 | 4.16(3.66) | 9 | 5.67(3.12) | 7 | 2.86(2.55) | 0.294 |
| Time 18 | 257 | 3.58(3.24) | 0 |  | 8 | 2.50(2.33) | 0.352 |
| Time 24  IES-R, mean (SD) | 242 | 3.43(2.92) | 0 |  | 12 | 4.42(3.21) | 0.254 |
| Time 3 | 295 | 14.41(13.23) | 7 | 4.43(5.83) | 7 | 12.29(15.50) | 0.132 |
| Time 6 | 283 | 8.33(9.47) | 8 | 7.50(8.00) | 11 | 6.18(4.77) | 0.735 |
| Time 13 | 271 | 6.04(7.30) | 9 | 5.00(3.87) | 7 | 5.43(3.74) | 0.892 |
| Time 18 | 255 | 4.72(6.57) | 0 |  | 8 | 1.75(1.91) | 0.204 |
| Time 24 | 242 | 3.59(5.90) | 0 |  | 12 | 3.08(3.78) | 0.770 |

^a^ Among the 321 family surrogates who participated in bereavement surveys, follow-up assessments were not due for 32 participants.

^b^ Bereavement 3 to 24 months, number of bereaved caregivers not participated in the previous assessments was 8, 7, 7, 11, and 1, respectively.

Each psychological-distress measurement was compared to the prior wave of assessment.
